# Smoking cessation changes basic metabolism, body weight, leptin and insulin levels, adipose tissue percentage, index of insulin resistance and index of insulin secretion

**DOI:** 10.1186/1617-9625-12-S1-A24

**Published:** 2014-06-06

**Authors:** Ioanna Nikoloutsou, Vasiliki Vasileiou, Eleni Litsiou, Stavroula Kolokytha, Aikaterini Tsoutsa, Spyros Zakynthinos, Paraskevi Katsaounou

**Affiliations:** 1 Pulmonary department-ICU, Εvaggelismos hospital, Athens, 10676, Greece; 2 Endocrinology Department, Alexandra hospital, Athens, 11528, Greece

## Background

It is known that nicotine increases energy expenditure, basic metabolism and has appetite-suppressing properties, which likely explains why smokers tend to have lower body weight than non-smokers and why smoking cessation is followed by weight gain up to 12 kgr (7kgr in median). Thus smoking cessation often has the disadvantage of increasing the risk of metabolic syndrome; fact that both decreases the benefits of smoking cessation and can discourage smokers (mostly women) from quitting. On the top of that, weight gain has been found to increase the risk of relapse, particularly among normal-weight or underweight women who report chronic dieting, but also among men. Varenicline is a nicotinic-cholinergic receptor partial agonist and therefore should share nicotine’s properties. Thus we believe that during its intake for three months in the process of smoking cessation basic metabolism will be sustained in levels comparable to these while smoking. During this period ex-smokers are also protected from withdrawal syndromes and thus capable of following psychological and physical activity counselling in order to sustain stable body weight. Quitting smoking is a process of changing life habits and is extremely difficult due to the addictive properties of nicotine. Post cessation weight gain decreases the benefits and leads to increased relaption rates. Varenicline improves succession and decrease relaption rates.

## Materials and methods

During the three months period of varenicline intake, we enrolled 15 smokers that visited our smoking cessation clinic and succeeded in quitting smoking. Eight used varenicline. Basic metabolism and body weight were measured before (BSC) and one month after smoking cessation (ASC).

## Results

Basic metabolism was 1418.4 ± 202 BSC and 1489.2±183 ASC in smokers that took varenicline and 1390.8 ± 220 BSC and 1250 ± 240 ASC in smokers that did not use the drug or any nicotine replacement therapy. Body weight was 79.4± 5 BSC and 81.3 ± 6 in the varenicline group and 74± 6 BSC and 78±5 ASC in the non varenicline group. Minimum weight increase in ex-smokers that use varenicline will maximize the profits of smoking cessation on the top of having a higher probability in succeeding to stop smoking. We also found that ex-smokers not only take extra weight, but this weight has the following consequences:

1. The percentage of adipose tissue is higher after smoking cessation (from 24.1% to 31.5%).

2. Both levels of leptin and leptin/fat mass were higher after smoking cessation (from 3318.156 pg/ml to 3384.1pg/ml).

3. Index of insulin resistance (HOMA IR) is higher after smoking cessation (HOMA IR: from 140.8 to 167.56)

4. Index of insulin secretion (HOMA B) is higher after smoking cessation (HOMA B: from 951.54 to 1022.75).

## Conclusions

Considering that tobacco and obesity are the two leading causes of preventable death, and since the relationship between tobacco smoke and metabolic syndrome is confirmed, we should help smokers to quit, increase the succession rates, decrease the relapse rates and maximize the benefits of smoking cessation by preventing post cessation metabolic syndrome.

